# Prognosis impact of clinical characteristics in patients with inoperable esophageal squamous cell carcinoma

**DOI:** 10.1371/journal.pone.0182660

**Published:** 2017-08-07

**Authors:** Ying Yang, Jun Jia, Zhiwei Sun, Feng Du, Jing Yu, Chuanling Liu, Yanjie Xiao, Xiaodong Zhang

**Affiliations:** Key laboratory of Carcinogenesis and Translational Research (Ministry of Education/Beijing), VIP-II Gastrointestinal Cancer Division of the Department of Medicine, Peking University Cancer Hospital and Institute, Haidian District, Beijing, China; Duke Cancer Institute, UNITED STATES

## Abstract

**Background:**

Patients with inoperable esophageal squamous cell carcinoma (ESCC) were not homogeneous and their outcomes were widely divergent. There was a lack of identified clinical factors related to prognosis; and there were no previous studies constructing prognosis score to predict survival and guide treatment.

**Methods:**

In this retrospective cohort study, twelve clinical characteristics of one hundred and twenty inoperable ESCC patients were collected at diagnosis and analyzed by Cox regression model. Various methods including univariate analysis, confounding adjusted multivariate analysis and model selection were applied to determine factors associated with poor prognosis; and prognosis score was built on established factors.

**Results:**

Four characters were identified as poor prognosis factors, including mid- and low-thoracic tumor (aHR = 2.20, 95% CI = 1.03, 4.72), abdominal and retroperitoneal lymph node metastasis (aHR = 1.62, 95% CI = 1.00, 2.64), albumin no more than 39g/L (aHR = 2.81, 95% CI = 1.24, 6.41) and hematogenous metastasis (aHR = 1.61, 95% CI = 0.97, 2.69). Patients were stratified into three groups by prognosis score, that was, good survival with none of four identified factors (score zero), poor survival with three to four factors (score three to four) and median with one to two factors (score one to two), survival of three groups were statistically different (*p*_*trend*_ = 0.020).

**Conclusion:**

Prognosis score based on selected clinical characteristics could predict survival among inoperable ESCC patients, which was critical for individualized treatment and central of precise medicine.

## Introduction

Esophageal cancer is a malignant disease seriously threatening human health and lives. In 2012, it was estimated that 455,800 new cases and 400,200 deaths of esophageal cancer occurred globally, which ranked the eighth in incidence and the sixth in mortality of cancers [1]. The mortality of esophageal cancer in China was the highest in the world. Chinese epidemiology data showed that there were estimated 477,900 incident cases and 375,000 deaths of esophageal cancer in 2015, which ranked the third in incidence and the fourth in mortality, respectively [2–4].

The major pathology types of esophageal cancer comprise of squamous cell carcinoma, adenocarcinoma and neuroendocrine carcinoma. In western countries and the US, approximately 50% of esophageal cancers are adenocarcinoma [5]; while in Asian countries especially in China, about 90% are squamous cell carcinoma and incidence is increasing [6, 7]. In general, the overall survival of esophageal cancer is as low as about 20% [8].

Surgery is the recognized best option to eradicate localized esophageal cancer. However, due to diverse impacts such as tumor location, comorbidities and patients’ will, only 15–20% of patients eventually receive surgical procedure, leaving quite a number of patients inoperable [8–12].

Although there is a lack of Chinese research data, taking international studies and our practice experience into account, it should be aware of that inoperable patients are the majority of esophageal squamous cell carcinoma (ESCC) with very poor prognosis in China; meanwhile, by our clinical observations, patients not receiving surgery are a heterogeneous population, with prominently different prognoses; and for the most benefit of patients, it is worthwhile to find factors predicting their survival and classify them by their prognoses, which would provide the basis and evidence for future precise treatment decisions.

To our knowledge, there were no widely accepted molecular markers related to ESCC survival, and even research on clinical factors in ESCC prognosis was little; thus in this study we tried to explore the association between the clinical features and survival in patients with inoperable ESCC, and also to stratify patients by their prognosis prediction factors, which might be of help to future treatment guidance.

## Methods

### Study population

A total of 196 esophageal cancer patients were treated in the VIP-II Gastrointestinal Cancer Division of the Department of Medicine, Peking University Cancer Hospital and Institute, from August 2012 to February 2016. Two patients rejected to attend the investigation and were excluded from our further analysis. The response rate was 98%. Of all included participants, 181 were squamous cell carcinomas, 11 were neuroendocrine carcinomas and 2 were adenocarcinomas. All were historically confirmed cases. Of the 181 ESCC patients, 61 received surgery and the other 120 patients did not because of advanced stages, tumor locations, comorbidities or refusal. All 120 inoperable patients were included in our study.

### Study design

It was a retrospective cohort study. The outcome was overall survival (OS), which was calculated as the number in months from the date of diagnosis to the date of death or the date of last follow-up on November 16th, 2016. The average follow-up was 40 months, ranging from 6 to 104 months. The exposures were twelve clinical and pathologic characteristics at diagnosis, including age, gender, comorbidities, and family history of cancers, multiple lesions, tumor location, differentiate grade, and lymphatic metastasis, hematogenous metastasis, and loss of weight, albumin and hemoglobin.

Some exposures were defined as follows. By endoscopy examinations, “multiple lesions” referred to more than one spot of malignant lesions that were separated by grossly normal tissues; and “tumor location” was measured in centimeters (cm) from the incisor to the superior boarder of tumor. 25cm was set as the cut-off and it was dealt as “non-applicable (NA)” if multiple lesions spread across the boundary of 25cm. Lymphatic and hematogenous metastasis was assessed by a variety of means such as esophagoscopy, esophageal ultrasonography, computed tomography (CT), Magnetic Resonance Imaging (MRI) or positron emission tomography-computed tomography (PET-CT) scans. We took “lymphatic metastasis” as four fields—cervical and supraclavicular lymph nodes, mediastinum lymph nodes, abdominal and retroperitoneal lymph nodes and other areas.

### Statistical analysis

Univariate analysis and multivariate analysis adjusting for confounders were used to identify prognosis related factors. We also applied model selection to opt for factors significantly related to survival. All survival analyses were performed by Cox proportional hazard regression model. All factors included in the regression model were tested for proportional hazard assumptions and no violations were detected. Integrated the results of univariate analysis, multivariate analysis and model selection into account, factors statistically significantly associated with poor prognosis were established as prognosis factors and the count of prognosis factors served as prognosis score. Furthermore, patients were stratified by their prognosis score; and the corresponding survival intervals were compared by the log-rank test and the survival curves were plotted by the Kaplan-Meier method. All statistical analyses were two-sided, and *p*<0.05 were treated as statistical significance. Results were presented by hazard ratios (HRs) and 95% confident intervals (95% CIs). All analyses and plots were conducted by SAS 9.4 (SAS Institute Inc., Cary, NC, USA).

The study was approved by the Institutional Review Board of Peking University Cancer Hospital and Institute.

## Results

The median age at diagnosis was 60 years of age, varying from 44 to 79 years. 78% of patients were male while 22% were female. 11% of patients were with multiple lesions. In terms of tumor location, about 70% located at 25cm and lower, that was mid- and low- thoracic ESCC. 87% of inoperable ESCC patients were with lymphatic metastasis, and among these patients 50% were with abdominal and retroperitoneal lymph node invasion. Hematogenous metastasis was found in 23% of patients, and further stratification revealed that 52% were lung metastasis, 22% were liver metastasis and 11% were bone metastasis. The mean albumin was 43.5g/L, with a range of 29.9g/L to 51.0g/L. The mean hemoglobin was 141.4g/L and the range was from 92.0 g/L to 175.0g/L. OS was from 0 to 63 months and its median was 16 months. By the date of last follow-up, 62% of patients died and 7% were loss of follow-up; and the others were still alive. As for treatment, 5% received solely radiotherapy and 24% received chemotherapy only; and 71% received multidisciplinary treatment that centered on chemoradiotherapy (CRT) and included other modalities such as radiofrequency ablation and “gamma knife”. The results were shown in Table 1.

**Table 1:** Clinical characteristics of inoperable ESCC patients (n = 120).

Characteristics	Counts	Percentage (%)
Age (years) (median) (range)	60 (44–79)	
Gender		
Male	93	78
Female	27	22
Past medical history		
No	54	45
Yes	66	55
Cancer family history		
No	86	72
Yes	34	28
Multiple lesions		
No	104	89
Yes	13	11
Tumor location		
<25cm	23	20
≥25cm	82	70
NA	12	10
Differentiation		
High	6	6
Median	58	56
Low	40	38
Lymphatic metastasis		
No	16	13
Yes	104	87
Abdominal and retroperitoneal lymph node metastasis	52	50
Other lymph node metastasis	52	50
Hematogenous metastasis		
No	92	77
Yes	27	23
Liver	6	22
Lung	14	52
Bone	3	11
Multiple	4	15
Loss of body weight		
No	47	45
Yes	58	55
Albumin (g/L) (mean±SD) (range)	43.5±3.94 (29.9–51.0)	
Albumin		
≤39g/L	11	10
>39g/L	95	90
Hemoglobin (g/L) (mean±SD) (range)	141.4±16.7 (92.0–175.0)	
Treatment		
Chemotherapy only	28	24
Radiotherapy only	6	5
Comprehensive therapy	82	71
OS (months) (median) (range)	16 (0–63)	
Follow-up		
Survival	38	32
Death	74	62
Loss of follow-up	8	7

Univariate analysis showed that tumor located at 25cm and lower (cHR = 2.39, 95% CI = 1.13, 5.05), lymphatic metastasis (cHR = 2.64, 95% CI = 1.06, 6.56), hematogenous metastasis (cHR = 1.71, 95% CI = 1.03, 2.84), and albumin≤39g/L (cHR = 2.46, 95% CI = 1.10, 5.51) were associated with poor prognosis. It was worth noting that abdominal and retroperitoneal lymph node metastasis (cHR = 1.76, 95% CI = 1.10, 2.83) was related to unfavorable prognosis. Next, we adjusted for age and gender whenever possible, the adjusted analyses revealed very similar results. Specifically speaking, mid- and low-thoracic tumor (aHR = 2.20, 95% CI = 1.03, 4.72), abdominal and retroperitoneal lymph node metastasis (aHR = 1.62, 95% CI = 1.00, 2.64) and low albumin (aHR = 2.81, 95% CI = 1.24, 6.41) were statistically significantly related to lower survival; while lymphatic metastasis (aHR = 2.36, 95% CI = 0.93, 5.98) and distant metastasis (aHR = 1.61, 95% CI = 0.97, 2.69) were marginally significant. All analyses were presented in Table 2.

**Table 2:** Association between clinical characteristics and prognosis of inoperable ESCC patients.

Characteristics	Univariate analysis		Adjusted analysis*	
	cHR (95% CI)	p value	aHR (95% CI)	p value
Age	0.99 (0.96, 1.02)	0.546	0.99 (0.96, 1.02)	0.509
Gender				
Male	1.00		1.00	
Female	0.61 (0.33, 1.12)	0.108	0.61 (0.33, 1.10)	0.104
Past medical history				
No	1.00		1.00	
Yes	1.08 (0.67, 1.74)	0.743	1.09 (0.66, 1.82)	0.738
Cancer family history				
No	1.00		1.00	
Yes	0.87 (0.51, 1.49)	0.605	0.93 (0.54, 1.60)	0.782
Multiple lesions				
No	1.00		1.00	
Yes	1.73 (0.87, 3.42)	0.116	1.65 (0.83, 3.28)	0.156
Tumor location				
<25cm	1.00		1.00	
≥25cm	2.39 (1.13, 5.05)	0.022	2.20 (1.03, 4.72)	0.042
Differentiation				
Low	1.00		1.00	
Median	1.22 (0.71, 2.08)	0.474	1.26 (0.74, 2.17)	0.394
High	1.18 (0.35, 3.94)	0.791	1.20 (0.36, 4.04)	0.771
Lymphatic metastasis				
No	1.00		1.00	
Yes	2.64 (1.06, 6.56)	0.037	2.36 (0.93, 5.98)	0.072
Abdominal and retroperitoneal lymph node metastasis				
No	1.00		1.00	
Yes	1.76 (1.10, 2.83)	0.019	1.62 (1.00, 2.64)	0.053
Hematogenous metastasis				
No	1.00		1.00	
Yes	1.71 (1.03, 2.84)	0.038	1.61 (0.97, 2.69)	0.068
Loss of body weight				
No	1.00		1.00	
Yes	1.25 (0.75, 2.07)	0.395	1.23 (0.74, 2.05)	0.415
Albumin				
>39g/L	1.00		1.00	
≤39g/L	2.46 (1.10, 5.51)	0.028	2.81 (1.24, 6.41)	0.014
Hemoglobin	1.01 (1.00, 1.03)	0.140	1.00 (0.99, 1.02)	0.551

*Adjusted for age and gender.

In addition, we put all twelve exposure variables that included age, gender, past medical history, and family history of cancer, multiple lesions, tumor location, and differentiation, lymphatic metastasis, hematogenous metastasis, and loss of weight, albumin and hemoglobin into one multivariate model, and applied stepwise selection. After model selection, hematogenous metastasis and low albumin entered into the final model; and the aHRs were 1.68 (95% CI = 1.00, 2.84) and 2.35 (95% CI = 1.05, 5.29), respectively.

Taking results of univariate analysis, adjusted analysis and model selection into consideration, tumor location≥25cm, abdominal and retroperitoneal lymph node metastasis, hematogenous metastasis and albumin≤39g/L were determined as poor prognosis factors. Each factor scored one point and a patient’s “prognosis score” was the sum of all factors, ranging from 0 to 4. The corresponding cases and median OS were shown in Table 3. The *p*_*trend*_ for groups was 0.012.

**Table 3:** Prognosis scores of inoperable ESCC patients.

Prognosis score	Cases	Percentage (%)	OS (median (range)) (months)
0	14	15	20 (7–48)
1	33	35	15 (3–51)
2	29	31	17 (2–63)
3	16	17	11 (0–44)
4	2	2	3.5 (3–4)

*p*_*trend*_ = 0.012

**Fig 1:**
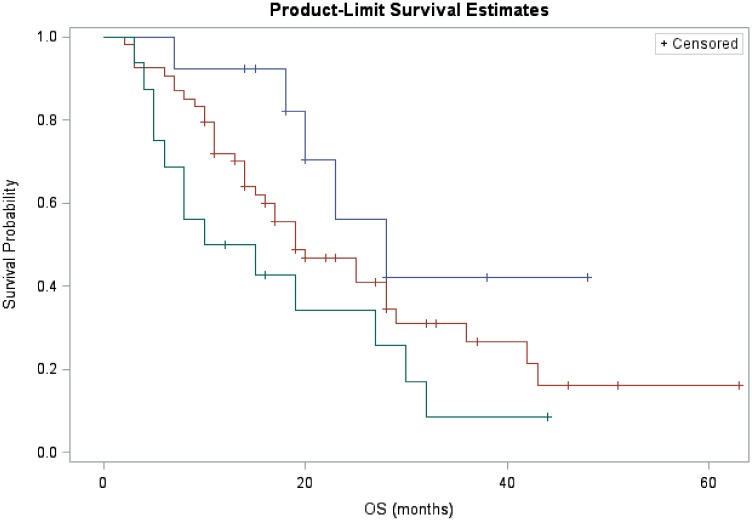
Kaplan-Meier curve of prognosis stratification in patients with inoperable ESCC. (blue line: good prognosis group; red line: median prognosis group; green line: poor prognosis group).

Besides, we stratified patients into three groups by their prognosis score—good with score zero, median with score one to two and poor with score three to four. 15% of patients were good prognosis with median OS of 20 months; and 19% were poor prognosis with median OS of 9 months. Results were listed in Table 4 and the corresponding Kaplan-Meier curves were presented in Fig 1. The three prognosis groups separated clearly and *p*_*trend*_ was 0.020.

**Table 4:** Prognosis stratification of inoperable ESCC patients.

Prognosis stratification	Prognosis score	Cases	Percentage (%)	OS (median (range)) (months)
Good	0	14	15	20 (7–48)
Median	1–2	62	66	16 (2–63)
Poor	3–4	18	19	9 (0–44)

*p*_*trend*_ = 0.020

In the end, we analyzed the association between treatment modalities and prognosis. By adjusting for age, gender, tumor location, and hematogenous metastasis, abdominal and retroperitoneal lymph node invasion and albumin level, comprehensive therapy, primarily CRT was significantly related to longer survival, compared with chemotherapy only (aHR = 0.27, 95% CI = 0.15, 0.51), Results were seen in Table 5.

**Table 5:** The relationship between treatment and prognosis.

Treatment model	Univariate analysis		Adjusted analysis*	
	cHR (95% CI)	p value	aHR (95% CI)	p value
Chemotherapy only	1.00		1.00	
Radiotherapy only	0.25 (0.08, 0.73)	0.011	0.34 (0.09, 1.26)	0.105
Comprehensive therapy	0.30 (0.18, 0.51)	<0.0001	0.27(0.15, 0.51)	<0.0001

*Adjusted for age, gender, tumor location, hematogenous metastasis, abdominal and retroperitoneal lymph node metastasis and albumin level.

## Discussion

In patients with inoperable ESCC, we built prognosis score based on four clinical factors including tumor located at 25cm and lower, abdominal and retroperitoneal lymph node invasion, hematogenous metastasis and albumin level no more than 39g/L; and found that survival of patients with none of the four factors was significantly better than those with three to four factors.

There had been an assortment of prognosis score systems derived from cancer features and host properties, such as the Physiologic and Operative Severity Score for the Enumeration of Mortality and Morbidity (POSSUM) [13], POSSUM adjusted for esophagogastric surgery [14], and Portsmouth POSSUM (P-POSSUM) [15], Glasgow Prognostic Score (GPS) [16], and Association of Coloproctology of Great Britain and Ireland scoring system [17]. Nevertheless, all those prognosis score models were to estimate post-operation comorbidity and mortality, and none of them were particularly for ESCC; thus their accuracy and application for prediction of inoperable ESCC long-term survival were equivocal.

A few of previous studies on the association between clinical characteristics and ESCC prognosis were inconsistent and inconclusive. For example, in thoracic ESCC patients, one study found that performance status, initial weight loss, lymph node stage, and serum C-reaction protein (CRP) level, cigarette smoking, and differentiation grade were related to prognosis [18]; and another reported that swallowing difficulty, cigarette smoking, number of invasive lymph nodes, and gastric cardia involvement were independent prognostic factors [19]. For another example, in patients treated with definitive CRT, a study reported that smoking history, poor differentiation and short progression free survival (PFS) were associated with unfavorable outcome [20].

In our study, we found that mid- and low-thoracic tumor, lymphatic metastasis especially abdominal and retroperitoneal lymph node invasion, distant metastasis and low albumin level were associated with poor prognosis in patients with inoperable ESCC. These findings were generally in accordance with clinical observations and consensus. Still, it should be noted that low albumin might be controversial. Several studies showed that hypoalbuminemia was related to more advanced T stage or decreased survival [21, 22]; while others revealed that decreased albumin solely was not associated with poor prognosis [23]; therefore, GPS containing CRP and albumin was introduced to estimate survival, which functioned well in some gastrointestinal cancers [24–26]. However, since CRP was easily influenced by kinds of diseases such as cardiovascular and cerebrovascular diseases, rheumaimmune systemic diseases and infection, we did not take it as a reliable indicator of malignant cancer survival. Therefore, we did not adopt GPS or CRP as our prognosis factors; and we believed that low albumin was poor prognosis related and was the only modifiable factor among all four. Early intervention to boost albumin level might improve patients’ long-term outcome.

Additionally, we analyzed the impact of treatment on survival. In the RTOG 85–01 randomized trial, concurrent CRT showed beneficial 5-year survival over radiotherapy alone; and toxicity was tolerable [27, 28]. In our study, we found that patients would be benefit more from comprehensive treatment primarily CRT, comparing to chemotherapy only. Since most ESCC patients were aged and quite a number were not eligible for surgery due to complications, CRT should be considered as an optimal alternative. Taking account of our results, for inoperable patients with good prognosis, comprehensive treatment should be considered as the priority, which would prolong survival even achieve cure; meanwhile, for patients with high prognosis score indicating poor survival, it might be inappropriate to administer aggressive treatment and further larger sample studies should be carried on to set up proper therapeutic models, with the aim of improving quality of life and lengthening survival to some extent.

As a retrospective cohort study, our analyses were affected by various kinds of biases. Known confounders like age and gender were adjusted in multivariate analysis and unknown confounding factors would pull results into either direction. Most interested characteristics in the study were objective measurements, so it was less likely that our results were influenced by recall bias; and measurement bias mainly pulled results into the null. Selection bias did exist and might either exaggerate or weaken the results. Besides, the sample size was small.

In the era of precision medicine, compared with other gastrointestinal tumors, research on ESCC that was one of the most important cancers in the Asian area, especially in China was falling far behind. Our study was the first to explore associations between clinical features and prognosis and to predict survival by the total of prognostic factors in Chinese patients with inoperable ESCC. With the consciousness that inoperable ESCC was not a homogeneous disease but without definite molecular markers for its prognosis, stratifying patients by clinical characteristics was also a means of precision medicine, which was critical and realistic in individualized treatment. Since all prognostic factors identified in our study were routinely measured at the time of diagnosis in clinical settings, prognosis score constructed in our study was practical and convenient. However, limited by sample size and inherent defects of an observational study, further larger prospective trials are warranted to confirm our findings.

## References

[1] Torre LA, Bray F, Siegel RL, Ferlay J, Lortet-Tieulent J, Jemal A. Global cancer statistics, 2012. CA: a cancer journal for clinicians. 2015;65(2):87–108. Epub 2015/02/06. doi: 10.3322/caac.21262 . 25651787 10.3322/caac.21262

[2] Chen W, Zheng R, Baade PD, Zhang S, Zeng H, Bray F, et al Cancer statistics in China, 2015. CA: a cancer journal for clinicians. 2016;66(2):115–32. Epub 2016/01/26. doi: 10.3322/caac.21338 . 26808342 10.3322/caac.21338

[3] Zhang H, Chen Z, Cheng J, Zhu X, Guo W, Hu A, et al The high incidence of esophageal cancer in parts of China may result primarily from genetic rather than environmental factors. Diseases of the esophagus: official journal of the International Society for Diseases of the Esophagus. 2010;23(5):392–7. Epub 2009/11/12. doi: 10.1111/j.1442-2050.2009.01020.x . 19903195 10.1111/j.1442-2050.2009.01020.x

[4] Zhou MG, Wang XF, Hu JP, Li GL, Chen WQ, Zhang SW, et al [Geographical distribution of cancer mortality in China, 2004–2005]. Zhonghua yu fang yi xue za zhi [Chinese journal of preventive medicine]. 2010;44(4):303–8. Epub 2010/07/27. . 20654141

[5] Vizcaino AP, Moreno V, Lambert R, Parkin DM. Time trends incidence of both major histologic types of esophageal carcinomas in selected countries, 1973–1995. International journal of cancer. 2002;99(6):860–8. Epub 2002/07/13. doi: 10.1002/ijc.10427 . 12115489 10.1002/ijc.10427

[6] Tran GD, Sun XD, Abnet CC, Fan JH, Dawsey SM, Dong ZW, et al Prospective study of risk factors for esophageal and gastric cancers in the Linxian general population trial cohort in China. International journal of cancer. 2005;113(3):456–63. Epub 2004/09/30. doi: 10.1002/ijc.20616 . 15455378 10.1002/ijc.20616

[7] Lu CL, Lang HC, Luo JC, Liu CC, Lin HC, Chang FY, et al Increasing trend of the incidence of esophageal squamous cell carcinoma, but not adenocarcinoma, in Taiwan. Cancer causes & control: CCC. 2010;21(2):269–74. Epub 2009/10/30. doi: 10.1007/s10552-009-9458-0 . 19866363 10.1007/s10552-009-9458-0

[8] Rustgi AK, El-Serag HB. Esophageal carcinoma. The New England journal of medicine. 2014;371(26):2499–509. Epub 2014/12/30. doi: 10.1056/NEJMra1314530 . 25539106 10.1056/NEJMra1314530

[9] Paulson EC, Ra J, Armstrong K, Wirtalla C, Spitz F, Kelz RR. Underuse of esophagectomy as treatment for resectable esophageal cancer. Archives of surgery (Chicago, Ill: 1960). 2008;143(12):1198–203; discussion 203. Epub 2008/12/17. doi: 10.1001/archsurg.143.12.1198 . 19075172 10.1001/archsurg.143.12.1198

[10] Dubecz A, Sepesi B, Salvador R, Polomsky M, Watson TJ, Raymond DP, et al Surgical resection for locoregional esophageal cancer is underutilized in the United States. Journal of the American College of Surgeons. 2010;211(6):754–61. Epub 2010/10/29. doi: 10.1016/j.jamcollsurg.2010.07.029 . 20980174 10.1016/j.jamcollsurg.2010.07.029

[11] Worni M, Martin J, Gloor B, Pietrobon R, D'Amico TA, Akushevich I, et al Does surgery improve outcomes for esophageal squamous cell carcinoma? An analysis using the surveillance epidemiology and end results registry from 1998 to 2008. Journal of the American College of Surgeons. 2012;215(5):643–51. Epub 2012/10/23. doi: 10.1016/j.jamcollsurg.2012.07.006 ; 23084493 10.1016/j.jamcollsurg.2012.07.006 PMC3479433

[12] Oezcelik A, Kaiser GM, Niebel W, Sleyman C, Treckmann JW, Sotiropoulos GC, et al Ten-year survival of esophageal cancer after an en-bloc esophagectomy. Journal of surgical oncology. 2012;105(3):284–7. Epub 2011/09/29. doi: 10.1002/jso.22096 . 21953648 10.1002/jso.22096

[13] Copeland GP, Jones D, Walters M. POSSUM: a scoring system for surgical audit. The British journal of surgery. 1991;78(3):355–60. Epub 1991/03/01. . 2021856 10.1002/bjs.1800780327

[14] Tekkis PP, McCulloch P, Poloniecki JD, Prytherch DR, Kessaris N, Steger AC. Risk-adjusted prediction of operative mortality in oesophagogastric surgery with O-POSSUM. The British journal of surgery. 2004;91(3):288–95. Epub 2004/03/03. doi: 10.1002/bjs.4414 . 14991628 10.1002/bjs.4414

[15] Nagabhushan JS, Srinath S, Weir F, Angerson WJ, Sugden BA, Morran CG. Comparison of P-POSSUM and O-POSSUM in predicting mortality after oesophagogastric resections. Postgraduate medical journal. 2007;83(979):355–8. Epub 2007/05/10. doi: 10.1136/pgmj.2006.053223 ; 17488869 10.1136/pgmj.2006.053223 PMC2600084

[16] Vashist YK, Loos J, Dedow J, Tachezy M, Uzunoglu G, Kutup A, et al Glasgow Prognostic Score is a predictor of perioperative and long-term outcome in patients with only surgically treated esophageal cancer. Annals of surgical oncology. 2011;18(4):1130–8. Epub 2010/10/29. doi: 10.1245/s10434-010-1383-7 . 20981494 10.1245/s10434-010-1383-7

[17] Yan J, Wang YX, Li ZP. Predictive value of the POSSUM, p-POSSUM, cr-POSSUM, APACHE II and ACPGBI scoring systems in colorectal cancer resection. The Journal of international medical research. 2011;39(4):1464–73. Epub 2011/10/12. doi: 10.1177/147323001103900435 . 21986149 10.1177/147323001103900435

[18] Nakatsu T, Motoyama S, Maruyama K, Usami S, Sato Y, Miura M, et al Tumoral CRP expression in thoracic esophageal squamous cell cancers is associated with poor outcomes. Surg Today. 2012;42(7):652–8. Epub 2012/02/22. doi: 10.1007/s00595-012-0147-3 . 22350301 10.1007/s00595-012-0147-3

[19] Lin CS, Chang SC, Wei YH, Chou TY, Wu YC, Lin HC, et al Prognostic variables in thoracic esophageal squamous cell carcinoma. The Annals of thoracic surgery. 2009;87(4):1056–65. Epub 2009/03/28. doi: 10.1016/j.athoracsur.2008.11.051 . 19324127 10.1016/j.athoracsur.2008.11.051

[20] Kim D-E, Kim U-J, Choi W-Y, Kim M-Y, Kim S-H, Kim M-J, et al Clinical Prognostic Factors for Locally Advanced Esophageal Squamous Carcinoma Treated after Definitive Chemoradiotherapy. Cancer Res Treat. 2013;45(4):276–84. doi: 10.4143/crt.2013.45.4.276 24454000 10.4143/crt.2013.45.4.276 PMC3893325

[21] Ataseven B, du Bois A, Reinthaller A, Traut A, Heitz F, Aust S, et al Pre-operative serum albumin is associated with post-operative complication rate and overall survival in patients with epithelial ovarian cancer undergoing cytoreductive surgery. Gynecol Oncol. 2015;138(3):560–5. Epub 2015/07/15. doi: 10.1016/j.ygyno.2015.07.005 . 26163893 10.1016/j.ygyno.2015.07.005

[22] Han L, Song Q, Jia Y, Chen X, Wang C, Chen P, et al The clinical significance of systemic inflammation score in esophageal squamous cell carcinoma. Tumour Biol. 2016;37(3):3081–90. doi: 10.1007/s13277-015-4152-1 . 26423404 10.1007/s13277-015-4152-1

[23] Forrest LM, McMillan DC, McArdle CS, Angerson WJ, Dunlop DJ. Comparison of an inflammation-based prognostic score (GPS) with performance status (ECOG) in patients receiving platinum-based chemotherapy for inoperable non-small-cell lung cancer. British journal of cancer. 2004;90(9):1704–6. Epub 2004/05/20. doi: 10.1038/sj.bjc.6601789 ; 15150622 10.1038/sj.bjc.6601789 PMC2409737

[24] Leitch EF, Chakrabarti M, Crozier JE, McKee RF, Anderson JH, Horgan PG, et al Comparison of the prognostic value of selected markers of the systemic inflammatory response in patients with colorectal cancer. British journal of cancer. 2007;97(9):1266–70. Epub 2007/10/10. doi: 10.1038/sj.bjc.6604027 ; 17923866 10.1038/sj.bjc.6604027 PMC2360467

[25] La Torre M, Nigri G, Cavallini M, Mercantini P, Ziparo V, Ramacciato G. The glasgow prognostic score as a predictor of survival in patients with potentially resectable pancreatic adenocarcinoma. Annals of surgical oncology. 2012;19(9):2917–23. Epub 2012/04/11. doi: 10.1245/s10434-012-2348-9 . 22488099 10.1245/s10434-012-2348-9

[26] Dutta S, Crumley AB, Fullarton GM, Horgan PG, McMillan DC. Comparison of the prognostic value of tumour and patient related factors in patients undergoing potentially curative resection of gastric cancer. American journal of surgery. 2012;204(3):294–9. Epub 2012/03/27. doi: 10.1016/j.amjsurg.2011.10.015 . 22444831 10.1016/j.amjsurg.2011.10.015

[27] al-Sarraf M, Martz K, Herskovic A, Leichman L, Brindle JS, Vaitkevicius VK, et al Progress report of combined chemoradiotherapy versus radiotherapy alone in patients with esophageal cancer: an intergroup study. Journal of clinical oncology: official journal of the American Society of Clinical Oncology. 1997;15(1):277–84. Epub 1997/01/01. doi: 10.1200/jco.1997.15.1.277 . 8996153 10.1200/JCO.1997.15.1.277

[28] Stahl M, Wilke H, Fink U, Stuschke M, Walz MK, Siewert JR, et al Combined preoperative chemotherapy and radiotherapy in patients with locally advanced esophageal cancer. Interim analysis of a phase II trial. Journal of clinical oncology: official journal of the American Society of Clinical Oncology. 1996;14(3):829–37. Epub 1996/03/01. doi: 10.1200/jco.1996.14.3.829 . 8622031 10.1200/JCO.1996.14.3.829

